# Can cryoprevention of oral mucositis be obtained at a higher temperature?

**DOI:** 10.1007/s00784-020-03765-9

**Published:** 2021-01-09

**Authors:** A. Mahdi, J. Stübner, M. Bergling, M. Jontell, J. Walladbegi

**Affiliations:** 1 Department of Oral and Maxillofacial Surgery, Institute of Odontology, The Sahlgrenska Academy, University of Gothenburg, Gothenburg, Sweden, Medicinaregatan 12D, PO Box 450, 405 30 Gothenburg, Sweden; 2 Department of Oral Medicine and Pathology, Institute of Odontology, The Sahlgrenska Academy, University of Gothenburg, Gothenburg, Sweden

**Keywords:** Healthy volunteers, Intraoral cooling device, Oral cryotherapy, Oral mucositis, Tolerability

## Abstract

**Objectives:**

Ice chips (IC) have successfully been used to prevent the development of chemotherapy-induced oral mucositis (OM). Although effective, IC entails several shortcomings and may open avenues for systemic infections as the water used may be contaminated by microorganisms, which may jeopardise the medical rehabilitation of an already immunosuppressed patient. This study aimed to investigate the efficacy and tolerability profile of a novel intraoral cooling device (ICD).

**Subjects and methods:**

In total, 20 healthy volunteers were enrolled in this randomised crossover study. Intraoral temperatures were registered using an IR camera, at baseline and following 30 and 60 min of cooling with the ICD, set to 8 °C or 15 °C. Following each cooling session, tolerability was assessed using a questionnaire.

**Results:**

A statistically significant difference in the intraoral temperature was observed using 8 °C compared with 15 °C, following both 30 (1.87 °C, *p* < 0.001) and 60 min (2.48 °C, *p* < 0.001) of cooling. Thus, the difference of the intraoral temperatures was less than the 7 °C difference between 8 °C and 15 °C. Furthermore, 60 min of cooling with 15 °C compared with 8 °C was better tolerated and preferred by 15 out of 20 participants (*p* < 0.001).

**Conclusion:**

Cooling was better tolerated when the ICD was set to 15 °C compared with 8 °C, although the difference in reduction of the intraoral mucosal temperature was marginal and may not affect cryoprevention of oral mucositis.

**Clinical relevance:**

The ICD has the potential to improve the care for patients with cancer at high risk of developing OM.

## Introduction

Cryotherapy (CT) has for long been used in various medical disciplines to manage a variety of life-threatening conditions, e.g. major stroke, severe traumatic brain injury and traumatic cardiac arrest [1]. CT has over the past decades further been applied in haematology/oncology to reduce the duration and severity of chemotherapy-induced oral mucositis (OM), a common debilitating sequel to haematopoietic stem cell transplantation (HSCT) [2–5].

OM refers to the painful, erythematous and ulcerative lesions in the oral mucosa which affects 40% of patients who receive standard dose of chemotherapy, and up to 80% of patients who are conditioned with high-dose chemotherapy in preparation for HSCT [6, 7]. Once established in the oral mucosa, OM can give rise to several distressing symptoms. These include oral pain, dysphagia and the need for parenteral nutrition, all of which may adversely affect the patient’s well-being. As a consequence, this may lead to increased need for analgesics, extended hospital visits [8–10] and entail increased healthcare costs [11, 12]. OM can further be a portal of entry for systemic infections which can lead to sepsis and death [13, 14].

**Fig. 1:**
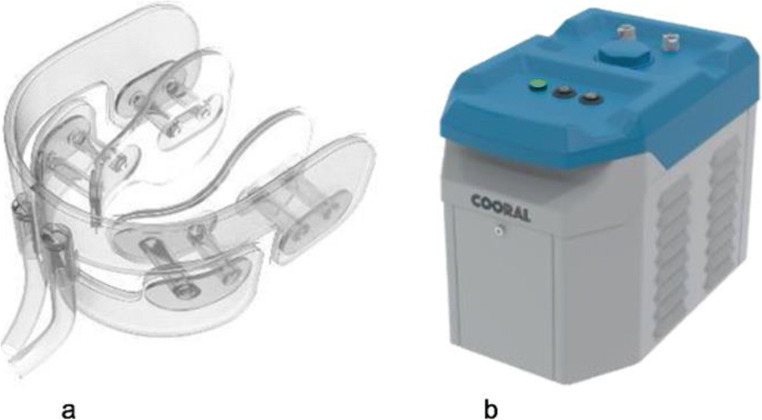
The Cooral® System. **a** Schematic illustration of the intraoral cooling device. **b** The portable thermostat unit. Reprinted and modified with permission from Walladbegi J., Gellerstedt M., Svanberg A., Jontell M. *Innovative intraoral cooling device better tolerated and equally effective as ice cooling*. *Cancer Chemother Pharmacol*. 2017 Nov; 80(5):965–72. (http://creativecommons.org/licenses/by/4.0/)

Current recommendations by the Multinational Association of Supportive Care in Cancer/International Society of Oral Oncology (MASCC/ISOO) for prevention of chemotherapy-induced OM include recombinant human keratinocyte growth factor-1 (KGF-1, palifermin), photobiomodulation and CT [4]. The latter comprises the largest proportion of studies and continues to be the best alternative of the three strategies [4]. Several studies, including randomised controlled trials, have consistently favoured the use of CT for prevention of chemotherapy-induced OM [5, 15, 16]. However, despite these positive observations, the use of ice cooling as a preventive method in clinical practice is limited. The reason for this may be that ice has a detrimental effect on the comfort level of the patients, e.g. by causing cold sensations, chills and shooting pain in the teeth, and thereby leading to poorer adherence to IC [17, 18]. In addition, the use of IC requires water of good quality, to avoid contamination by microorganisms, which may have an unfavourable effect for an already immunosuppressed patient. Therefore, there have been demands in the literature to improve the use of cooling devices for cryoprevention (CP) [18]. In response to this, an intraoral cooling device (ICD; Fig. 1a), which can be set to operate at different temperatures, has been developed. In a previous study [17], we observed that the ICD was better tolerated than IC and that the two cooling methods were equally effective with regard to intraoral temperature reduction. It is therefore of interest to elucidate if higher temperatures than those previously obtained by IC could enhance tolerability without compromising the intraoral temperature necessary to prevent chemotherapy-induced OM.

## Subjects and methods

### Subjects

The subjects comprised 20 (5 men and 15 women) healthy dental students, mean age of 23 years (SD = 1 year). To be eligible for participation in this study, the subjects had to meet all of the following inclusion criteria: (i) willing and able to provide informed written consent, (ii) age ≥ 18 years, (iii) no medical diagnoses established by physician, (iv) no medication with any impact on the cardiovascular system and (v) no use of tobacco or oral tobacco products or snuff. Subjects were excluded from the study (i) if the investigators observed any intraoral mucosal lesions which could interfere with the study protocol and (ii) if they deviated from the normal values for BMI and basic haemodynamics (heart rate, systolic- and diastolic blood pressure). All the subjects were recruited from the Institute of Odontology, The Sahlgrenska Academy, University of Gothenburg, Gothenburg, Sweden.

### Study design

This study was an experimental, double-blinded, randomised crossover trial in healthy volunteers to compare the efficacy and tolerability profile of the ICD set at two different temperatures (8 °C and 15 °C). All subjects attended two separate cooling sessions of 60 min each, with a minimum of 24 h apart. Half (*n* = 10) of the subjects started cooling with the ICD set 8 °C and crossed over to 15 °C; the other half (*n* = 10) carried out the procedure in the reverse order. A free online software tool for randomisation (www.randomizer.org) was employed to randomly assign the subjects to the order in which the two procedures were to be commenced. All the cooling sessions were conducted during spring of 2018 at the Department of Oral Medicine and Pathology, Institute of Odontology, the Sahlgrenska Academy, University of Gothenburg, Gothenburg, Sweden.

### Endpoints

The primary endpoint was:

- (i) The difference in mean temperature reduction (°C) after 60 min of cooling between the ICD set to 8 °C and 15 °C, respectively.

The secondary endpoints were:

- (ii-a) The difference in mean temperature reduction (°C) after 30 min of cooling between the ICD set to 8 °C and 15 °C, respectively.
- (ii-b) The difference in mean temperature reduction (°C) between 30 and 60 min of cooling with the ICD set to 8 °C and 15 °C, respectively.
- (ii-c) The tolerability of cooling after 60 min with the ICD set to 8 °C and 15 °C, respectively, by responding to the question, “Which of the two cooling sessions did you tolerate better?”
- (ii-d) Adverse events reported for cooling with the ICD set to 8 °C and 15 °C (descriptively).

### Instruments

#### Intraoral cooling device

The ICD (Cooral® Mouth Device; Fig. 1a) was provided by a Swedish medical technology company (BrainCool AB, Lund, Sweden). The ICD is composed of soft plastic material (polyolefin polymer compound based on ethylene-vinyl acetate copolymer) that comprises closed conduits with continuously circulating water to evenly distribute the cooling medium to the following intraoral locations: buccal mucosae, lips, floor of the mouth, tongue, gingiva and hard palate. The water was delivered via a portable thermostat unit (Cooral® System; Fig. 1b), which can be set to operate at fixed temperatures between 6 °C and 22 °C (± 2 °C). The thermostat unit was connected to the ICD, which was available in two sizes (small and medium), by tubes that allow a flow rate of 0.25 (± 0.1) L/min [17, 19].

#### Thermographic camera and image analysis tool

The FLIR E60 (bx) (FLIR Systems Inc., Wilsonville, OR, USA) is a thermographic camera with a level of resolution (320 × 240 pixels) that facilitates detection of small temperature discrepancies of 0.05 °C. The thermographic camera and its associated FLIR tools software were used to illustrate and numerically quantify temperature changes in the intraoral mucosa.

#### Questionnaire

A study-specific questionnaire comprising a total of seven (*n* = 7) questions was used to assess the non-parametrical endpoints of this study, i.e. the secondary endpoints addressing tolerability, and adverse events related to any of the two cooling sessions. The tolerability was addressed by the question “Which of the two cooling sessions did you tolerate better?”. The questionnaire was further designed to collect data regarding adverse events and to obtain explanations as to why cooling was interrupted if the cooling session was incomplete, i.e. not endured for 60 min. There was also space to share other comments related to discomfort, in running text. The questionnaire was adopted from the previous study investigating the ICD [17]. It was however translated into Swedish, by a professional language editor and subsequently amended to suit the purpose of this study. Prior to the study start, all questions and response alternatives were tested and discussed with an independent group of participants (*n* = 5). In this manner, the questionnaire was face-validated to ensure that the questions were interpreted as intended.

### Procedures and data collection

All the procedures were performed in the same examination office (ambient temperature 22 ± 2 °C) to maintain standardisation in the experimental design because the intraoral temperature may be susceptible to a cold environment. Prior to consent to study participation, each subject received extensive verbal and written information in Swedish regarding the purpose of the study, study procedures and any risks associated with using the ICD. They were then given the opportunity to ask questions related to the study before giving written informed consent. Subsequently, medical history was gathered, height and weight were registered for BMI calculation and basic haemodynamics were measured in left upper arm with the subject in sitting position, using a digital blood pressure cuff (Omron, HigashiNoda, Osaka, Japan). An intraoral mucosal examination was carried out by the investigators (JS, MB). This was to ensure that the subjects displayed a healthy intraoral mucosa. Eligible participants were then assigned to the first of a total of two cooling sessions, using the software tool for randomisation. The subjects were blinded to the cooling temperatures and were requested to refrain from eating and drinking for at least 30 min prior to each cooling session.

Prior to the first cooling session, hands-on demonstrations were set up to for the subjects to get acquainted with the ICD/cooling procedure and to find an appropriate size of the ICD that would be used throughout the study, i.e. small or medium. Upon cooling, the participants were asked to seat themselves in a chair in an upright or supine, 70° head-up position, if desired. The ICD was self-inserted by the participants under surveillance and screened by the investigators to verify a good adherence to the oral mucosa. At baseline and following 30 and 60 min of cooling, with the ICD set to either 8 °C or 15 °C, temperatures were recorded in a systematic order in eight (*n* = 8) intraoral locations: right buccal mucosa, left buccal mucosa, upper labial mucosa, lower labial mucosa, dorsal tongue, ventral tongue, floor of the mouth and hard palate, using the FLIR thermographic camera. After adjusting proper focus and contrast, settings were performed to maximise the location of interest in the field of view. All image recordings were performed by the same two investigators (JS, MB) without any preparations of the oral mucosa, other than asking the subject to swallow to remove excess saliva or debris from the operating field. Two regular dental mirrors made of stainless steel were used to access the site of interest and facilitate temperature imaging.

Before crossing over to the second cooling session, with at least 24 h apart, each subject was asked to complete the first part of the questionnaire which was related to any adverse events of the recently completed cooling session. The same procedure was repeated during the second cooling session. Ultimately, when both cooling sessions were completed, the participants were asked to declare which of the two cooling sessions they tolerated better.

For each of the eight (*n* = 8) intraoral locations, one steady image was recorded and assigned with a specific trial number, before data was exported, computer stored and analysed blindly by two independent investigators (AM, JW). The mean intraoral temperature for each subject was then calculated using FLIR tools software and subsequently grouped for the statistical analysis. The investigators had been previously calibrated using a subset of test images.

### Statistical analysis

A post hoc analysis, based on the results from the primary endpoint, showed a power of 99.9%. The power was calculated using a two-sided paired samples Student’s *t* test, sample size (*n* = 20), mean difference (*x̄* = 2.48 °C), standard deviation (SD = 1.71 °C) and an α-significance level of 0.05, employing G*power version 3.1.9.4 (University of Düsseldorf, Germany).

Normality assumption was controlled for all quantitative data using the Shapiro-Wilk test and a Gaussian distribution was confirmed for the tested variables. The descriptive data was presented with means (*x̄*) and standard deviations (SD). The primary endpoint, i.e. the difference in mean temperature reduction (°C) after 60 min of cooling between the ICD set to 8 °C and 15 °C, was analysed using a mixed model ANOVA. The secondary endpoints, i.e. the difference in mean temperature reduction (°C) after 30 min of cooling between the ICD set to 8 °C and 15 °C and the difference in mean temperature reduction between 30 and 60 min of cooling with the ICD set to 8 °C and 15 °C, respectively, were analysed in the same manner as the primary endpoint. The secondary endpoint of tolerability, expressed by “Which of the two cooling sessions did you tolerate better?”, was calculated with a McNemars test. The secondary endpoint related to adverse events was presented descriptively. A *p* value ≤ 0.05 was considered statistically significant. The calculations were employed using the IBM SPSS Statistics software package (IBM SPSS Statistics version 25, IBM, Armonk, NY).

### Ethical considerations

All the procedures in this study involving human participants were performed in accordance with the ethical principles established in the WMA Declaration of Helsinki (Fortaleza, October 2013). The study was also reviewed and approved by the Department of Oral Medicine and Pathology, Institute of Odontology, Sahlgrenska Academy, University of Gothenburg, Gothenburg, Sweden, which was a request by The Swedish Ethical Review Authority. The Swedish Ethical Review Authority itself did not consider an ethical application necessary. All participants received information concerning the study and were further informed about the rights to withdraw consent to participate at any given time without reprisal. Informed written consent was obtained from all participants.

## Results

All subjects enrolled in this study were healthy and reported no medical conditions. Table 1 provides a summary of the subject characteristics, BMI (kg/m^2^), basic haemodynamics (heart rate, bmp; systolic and diastolic blood pressure, mmHg; mean arterial pressure, mmHg) and procedural times in the present study. In total, 40 cooling sessions were completed, and all participants endured the 60 min of cooling with the ICD set to both temperatures. Furthermore, all questionnaires were completed and a total of 960 intraoral thermographic images were captured during this study.

**Table 1:** Summary of baseline datasets for subject characteristics, basic haemodynamics and procedural times. Quantitative parameters are presented as mean ± SD

Subject characteristics		
Gender	(F:M)	15:5
Age	(years)	23 ± 1
Mass	(kg)	66 ± 10
Height	(m)	1.7 ± 0.8
BMI	(kg/m^2^)	22.7 ± 2.4
Basic haemodynamics		
HR	(bpm)	61 ± 8
SBP	(mmHg)	123 ± 13
DBP	(mmHg)	81 ± 9
MAP	(mmHg)	95 ± 10
Procedural times		
Subj. charact./haemodyn.	(min)	10 ± 1
T0/T1/T2 measurements	(min)	5^a^ ± 1
ICD^8 °C^	(min)	60 ± 0
ICD^15 °C^	(min)	60 ± 0
Total experiment time	(min)	145 ± 1

*BMI* body mass index, *HR* heart rate, *bpm* beats per minute, *SBP* systolic blood pressure, *DBP* diastolic blood pressure, *MAP* mean arterial pressure, *mmHg* millimetres of mercury, *T0* baseline, *T1* 30 min, *T2* 60 min, *ICD*^*8 °C*^ intraoral cooling device (ICD) set to 8 °C, *ICD*^*15 °C*^ ICD set to 15 °C, *SD* standard deviation

^a^Each measurement

**Fig. 2:**
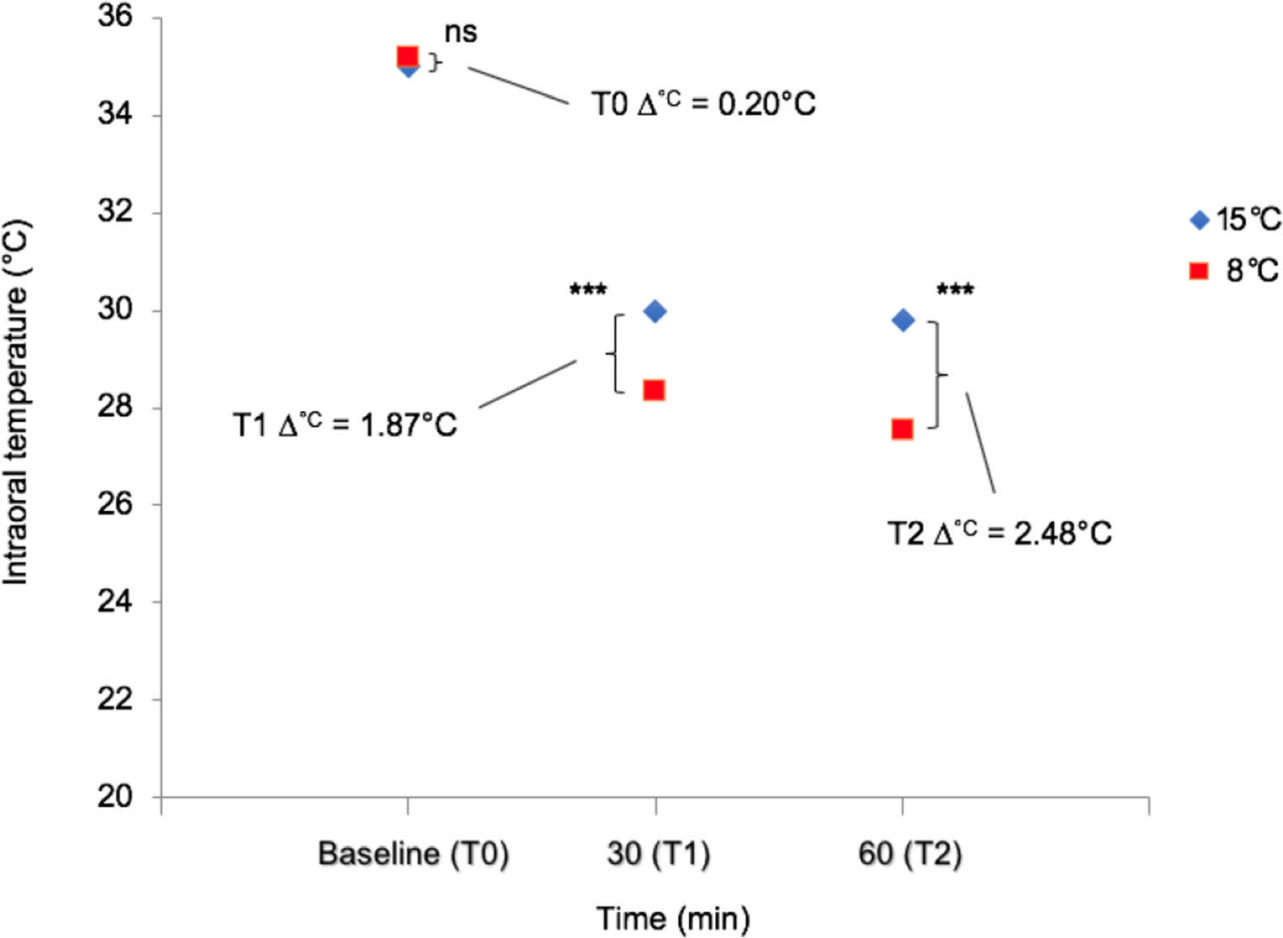
Comparison between intraoral temperatures at baseline (T0), 30 min (T1) and 60 min (T2), with the intraoral cooling device set to 8 °C and 15 °C, respectively; ns, not significant; ***p < 0.001

At baseline, prior to the cooling sessions with the ICD set to either 8 °C or 15 °C, mean intraoral temperatures were 35.2 °C and 35.0 °C, respectively (Fig. 2). This difference of 0.2 °C was not statistically significant.

Following 60 min of cooling, with the ICD set to either 8 °C or 15 °C, a statistically significant difference between the two observations was demonstrated. In the cooling sessions using 8 °C, a mean temperature reduction of 7.69 °C was observed, whereas the corresponding temperature reduction at the same follow-up period when using 15 °C was 5.21 °C (Table 2). Thus, the evaluation of the primary endpoint showed a difference in mean temperature reduction of 2.48 °C between the cooling sessions (*p* < 0.001; 95% CI 1.30 to 3.40) (Fig. 2).

**Table 2:** Mean intraoral temperature reduction after 30 and 60 min of cooling with the intraoral cooling device (ICD) set to 8 °C and 15 °C, respectively. Half of the subjects (*n* = 10) started cooling with the ICD set to 8 °C and crossed over to 15 °C

Subject nr.	Temp. reduction-30 min		Temp. reduction-60 min	
	8 °C	15 °C	8 °C	15 °C
1	6.43	3.93	7.28	4.93
2	3.38	3.24	6.48	3.50
3	7.80	5.30	8.30	6.36
*4*	9.64	6.51	8.88	5.60
*5*^a^	3.86	3.83	5.41	3.84
6^a^	6.60	5.26	7.39	7.58
7	9.01	8.64	11.45	8.34
*8*	7.73	6.62	7.25	5.94
9	5.94	4.54	7.85	4.35
10^a^	7.64	4.31	8.85	3.89
*11*^a^	6.31	3.06	5.48	2.70
*12*	8.43	6.95	9.59	6.61
13	6.85	4.64	8.34	4.91
*14*	9.03	4.35	10.55	4.56
15	5.75	4.69	8.08	4.93
*16*^b^	9.28	3.01	9.66	4.90
*17*	5.80	4.70	5.56	5.64
*18*	6.43	4.19	5.14	3.20
*19*	6.90	5.83	6.26	6.14
20	5.11	6.82	6.04	6.35
Mean	6.89	5.02	7.69	5.21
SD	1.71	1.48	1.80	1.45

The other half (*n* = 10; italicised) carried out the procedure in the reverse order. In total, 15 out of 20 subjects preferred cooling with the ICD set to 15 °C. Four (*n* = 4; marked with ^a^) had no preference with regard to cooling temperature, and the remaining subject (*n* = 1; marked with ^b^) preferred cooling with 8 °C

The statistical analysis for the secondary endpoint, i.e. the difference in mean temperature reduction (°C) after 30 min of cooling between the ICD set to 8 °C or 15 °C, also revealed a statistically significant difference between the cooling sessions (Fig. 2). Mean temperature reduction in the cooling sessions using 8 °C was 6.89 °C, whereas the same figure for the cooling session using 15 °C was 5.02 °C (Table 2). Thus, there was a mean difference of 1.87 °C (*p* < 0.001; 95% CI 0.73 to 2.60) (Fig. 2).

Following 30 and 60 min of cooling with the ICD set to 8 °C showed a mean temperature reduction of 6.89 °C and 7.69 °C, respectively (Table 2). The difference in mean temperature reduction of 0.8 °C was not statistically significant (Table 3). The same analysis, i.e. after 30 and 60 min for 15 °C, was 5.02 °C and 5.21 °C, respectively (Table 2). This difference of 0.19 °C did not reach statistical significance (Table 3).

**Table 3:** Difference in mean temperature reduction at 30 and 60 min, with the intraoral cooling device (ICD) set to 8 °C and 15 °C, respectively

Cooling session	Mean diff. (°C)	*p* value	95% CI	
ICD^8 °C^ T1 vs. T2	0.80	0.154 ns	− 0.31	1.90
ICD^15 °C^ T1 vs. T2	0.19	0.641 ns	− 0.64	1.02

*ICD*^*8 °C*^ ICD set to 8 °C, *ICD*^*15 °C*^ ICD set to 15 °C, *T1* 30 min, *T2* 60 min, *ns* not significant

The questionnaire was completed by all subjects (*n* = 20), and the responses to “Which of the two cooling sessions did you tolerate better?”, were as follows: one (*n* = 1) individual preferred cooling with the ICD set to 8 °C, four (*n* = 4) had no preference (shared in running text) and rated the two temperatures as equally tolerable and 15 participants preferred cooling with the ICD set to 15 °C. Thus, 15 of the 20 subjects (75%) preferred cooling with the ICD set to the higher cooling temperature. This difference was statistically significant (*p* < 0.001; 95% CI 50.9 to 91.3).

Concomitant registration for subject-reported adverse events is presented in Table 4. Rubbing discomfort was the most commonly reported adverse event in both cooling sessions, for 8 °C (*n* = 12) and for 15 °C (*n* = 12). In addition, in the cooling sessions using the ICD set to 8 °C, the following were reported: cold (*n* = 8), difficulties swallowing (*n* = 8), and poor fit (*n* = 7). The corresponding figures for the cooling sessions using the ICD set to 15 °C was cold (*n* = 2), difficulties swallowing (*n* = 9) and poor fit (*n* = 8).

**Table 4:** Subject reported adverse events for the two tested temperatures

Adverse events	8 °C (*n*)	15 °C (*n*)
Cold	8	2
Numbness	3	0
Bad taste	3	3
Headache	0	1
Teeth sensation	5	1
Pain	6	5
Poor fit	7	8
Nausea	1	1
Vomiting sensation	5	3
Difficulties swallowing	8	9
Rubbing discomfort	12	12
Other discomforts	5	2
Hypersalivation	3	0
Jaw discomfort	1	2
Cold tubes	1	0

## Discussion

The intraoral mucosa is a highly vascularised and innervated structure, which serves to protect the underlying tissues from mechanical stress, microorganisms and harmful toxins [20]. Even minor disruptions of these functions can seriously jeopardise the integrity of the oral mucosa and pose an increased risk to immunocompromised patients. OM constitutes such a risk which may lead to a potentially fatal condition. Fortunately, the development of chemotherapy-induced OM can be effectively inhibited by CP. In addition to reducing severity and duration of OM, CP has also been observed to decrease the number of days with total parenteral nutrition [16, 21], need for analgesics [15, 16, 22], length of hospitalisation [21] and pain related to OM [16]. Regrettably, IC has not been adopted as expected. This is surprising as the two leading international organisations—MASCC/ISOO—have for long recommended CP for management of OM [4]. Thus, numerous patients are at risk of suffering from the adverse effects of OM. One possible explanation for this neglection is the obvious infectious risk making IC from contaminated tap water and thereby threaten the medical rehabilitation of an already immunosuppressed patient [23–25]. This was the rationale for the development of a novel ICD [17].

The primary endpoint of this study measured the difference in mean temperature reduction after 60 min of cooling between the ICD set to 8 °C or 15 °C. Our results demonstrated a statistically significant difference at the follow-up between the two temperatures tested. However, although a statistically significant difference was seen, the clinical relevance remains questionable. Most likely, the additional temperature reduction of approximately 2 °C will not provide superior benefits in CP of OM.

As part of the secondary endpoint, the mean temperature reduction between the ICD set to 8 °C or 15 °C was also evaluated after 30 min of cooling and the obtained data for the respective temperatures was compared with the corresponding data following 60 min of cooling. This was carried out in order to investigate during what time interval, 0–30 min or 31–60 min, the most significant temperature reduction occurs, and if longer cooling times provide additional effects of relevance with regard to temperature reduction. Similar to the results for the primary endpoint, there was a statistically significant difference after 30 min of cooling between the ICD set to 8 °C or 15 °C. However, the difference was of a smaller magnitude and therefore probably of even less clinical significance.

As expected, the greatest effect in temperature reduction, regardless of the two temperatures tested, occurred within the first-time interval. The result indicates that 30 min of cooling with the ICD is sufficient to achieve a steady-state temperature in the intraoral mucosa. However, it is not evident from our study when during the initial 30 min the steady-state temperature is achieved. To establish this time-point is a topic of interest to be investigated in future studies. There are two main reasons for such a study to be conducted. Firstly, if shorter cooling sessions are sufficient to achieve the steady-state temperature, tolerability is most likely enhanced which would be advantageous. Secondly, present clinical studies differ markedly with respect to when to start cooling prior to the chemotherapy.

Surprisingly, in the majority of clinical studies and in a comprehensive literature review by the Cochrane collaboration, no or few adverse events have been reported for CT using IC [13, 16, 26]. However, these results are not in line with our previous study [17], where several adverse events, e.g. cold, headache, and teeth sensations, were reported in the sessions using IC. This study further showed that the novel cooling method which employs higher cooling temperatures (8 °C) than IC was significantly better tolerated and was preferred over IC by 80% of the participants. Given this, it was of interest as part of the secondary endpoints to assess the tolerability profile of higher cooling temperatures than those previously reported as well to assess adverse events for the ICD. In the present study, it was shown that higher cooling temperature was better tolerated by the majority of the subjects and that self-reported adverse events were more frequently reported in the sessions with the ICD set to 8 °C. These were mainly attributed to temperature-related factors, which is not surprising as lower temperatures are accompanied by increased discomfort [17]. However, in both sessions, regardless of cooling temperature used, adverse events related to the design of the ICD were frequently reported. Thus, it is essential to modify the design of the ICD prior to implementation in clinical settings.

However, it is worth mentioning that despite the complaints, the majority of the subjects reported that cooling did not cause problems to such extent that they could not undertake other activities during the time of cooling. This suggests that the overall perception of the ICD was acceptable. Given these results and the fact that there is room to further improve the design, the ICD can be considered for CP of OM in future clinical trials. This is particularly true in areas where the risk of contamination from water of poor quality is substantial [23–25].

This study has some limitations that should be acknowledged. Firstly, a common weakness of crossover trials is the carryover effect. This is, however, unlikely to have affected the results of this study as a washout period of at least 24 h between the cooling sessions was imposed. Secondly, the thermographic camera is not specifically designed by the manufacturer for the purpose of this study. However, given that these potential limiting factors were similar in both intervention groups, it can be assumed that it has influenced the outcome to similar extent. It is noteworthy that the subjects in this study were dental students with no general health conditions or compromised oral health status. This is likely not the scenario in clinical settings with cancer patients, which may complicate the use of the ICD.

## Conclusion

In conclusion, this study demonstrates that intraoral cooling using the ICD with a temperature of 15 °C is better tolerated than 8 °C but displays inferior capacity in temperature reduction of the oral mucosa. However, to elucidate whether this discrepancy of approximately 2 °C is of clinical importance, the optimal temperature for prevention of OM needs to be identified.

## Data Availability

Data and material are available.
